# Tacrolimus dose adjustment is not necessary in dose to dose conversion from a twice daily to a prolonged release once daily dose form

**DOI:** 10.1038/s41598-022-14317-4

**Published:** 2022-06-16

**Authors:** Kanitha Tiankanon, Stephen J. Kerr, Siriwan Thongthip, Suwasin Udomkarnjananun, Pimpayao Sodsai, Athaya Vorasittha, Kamol Panumatrassamee, Kullaya Takkavatakarn, Kriang Tungsanga, Somchai Eiam-Ong, Kearkiat Praditpornsilpa, Yingyos Avihingsanon, Natavudh Townamchai

**Affiliations:** 1 Division of Nephrology, Department of Medicine, Faculty of Medicine, Chulalongkorn University and King Chulalongkorn Memorial Hospital, Bangkok, Thailand; 2 Biostatistics Excellence Centre, Faculty of Medicine, Chulalongkorn University, Bangkok, Thailand; 3 Maha Chakri Sirindhorn Clinical Research Center, Chulalongkorn University, Bangkok, Thailand; 4 Excellence Center for Solid Organ Transplantation, King Chulalongkorn Memorial Hospital, Bangkok, Thailand; 5 Renal Immunology and Renal Transplant Research Unit, Department of Medicine, Faculty of Medicine, Chulalongkorn University, Bangkok, Thailand; 6 Center of Excellence in Immunology and Immune-Mediated Diseases, Department of Microbiology, Faculty of Medicine, Chulalongkorn University, Bangkok, Thailand; 7 Department of Surgery, Faculty of Medicine, Chulalongkorn University and King Chulalongkorn Memorial Hospital, Bangkok, Thailand; 8 Division of Urology, Department of Surgery, Faculty of Medicine, Chulalongkorn University and King Chulalongkorn Memorial Hospital, Bangkok, Thailand

**Keywords:** Transplant immunology, Immunology

## Abstract

Twice daily TAC (BID TAC) and prolonged released once daily dose tacrolimus (OD TAC) have different pharmacokinetic (PK) profiles in kidney transplant (KT) recipients. Precise dose adjustment recommendations when converting from BID TAC to OD TAC remain inconclusive. A single center, PK study was conducted in stable KT recipients taking constant doses of TAC, mycophenolic acid, and prednisolone. The area under the concentration–time curve (AUC) 0–24 and C_trough_ were measured before and 4 weeks after 1:1 conversion from BID TAC to OD TAC without subsequent dose adjustment. A 90% confidence interval (CI) of geometric mean ratio (GMR) of OD TAC/BID TAC within the range of 0.9–1.11 was utilized to indicate equivalence of the narrow therapeutic index drugs. The roles of CYP3A5 genotypic polymorphism on PK parameters were also assessed. There were 20 patients with median time since transplantation of 18 months. The mean of CKD-EPI eGFR was 60.7 ± 16.43 mL/min/1.73 m^2^. The median total daily TAC dose of 0.058 mg/kg/day. The geometric means (%CV) of AUC_0-24_ of OD and BID TAC were 205.16 (36.4%) and 210.3 (32.5%) ng/mL × h, respectively, with a GMR of 0.98 (90%CI 0.91–1.04). The geometric means (%CV) of C_trough_ of OD TAC and BID TAC were 5.43 (33.1%) and 6.09 (34.6%) ng/mL, respectively. The GMR of C_trough_ was 0.89 (90%CI 0.82–0.98), which was below 0.9. The newly calculated target C_trough_ level of OD TAC was 4.8–6.2 ng/mL. The best abbreviated AUC_0-24_ was AUC = 0.97(C0) + 5.79(C6) + 18.97(C12) − 4.26. The GMR AUC_0-24_ was within the range of 0.9–1.11 irrespective of CYP3A5 genotypic polymorphism while the GMR of C_trough_ was below 0.9 only in the CYP3A5 expressor patients. The 1:1 conversion from BID TAC to OD TAC without subsequent dose adjustment provided similar AUC_0-24_ regardless of CYP3A5 genotypic polymorphism. However, the C_trough_ was lower in the CYP3A5 expressor group. Therefore, it is not necessary to routinely increase the OD TAC dose after conversion.

**Trial registration:** Thai Clinical Trials Registry (TCTR20210715002).

## Introduction

Tacrolimus (TAC) is one of the main immunosuppressive drugs used to prevent allograft rejection after kidney transplantation (KT). There are two available oral forms: (1) Prograf^®^, a TAC that is administered twice daily (BID TAC), and (2) Advagraf^®^, a newer prolonged released once daily (OD TAC). Even though the formulation and pharmacokinetics (PK) of both drugs are different, yet they both have comparable efficacy in preventing rejection and have similar adverse event rates^1–3^. OD TAC is more convenient to administer and improves patient compliance^4^. However, in clinical practice, PK monitoring of the TAC levels is mandatory because TAC has a narrow therapeutic index^5^. Aside from that, the PK results have shown that there are high inter-patient variabilities^5^. Individual TAC PK can be affected by several factors, including *CYP3A5* genotypic polymorphism^5–7^.

Measuring area under the concentration–time curve (AUC) is the gold standard for monitoring TAC exposure and accurately represents the total daily exposure for each patient while the trough level concentration (C_trough_) is more practical and preferred in clinical practice even though it only provides TAC exposure just before the morning dose. Scientifically, to maintain the same level of TAC exposure between BID TAC and OD TAC, both drugs should have an equivalent level of AUC_0-24_ rather than C_trough_. Despite this crucial pharmacokinetic knowledge, several earlier PK studies pertaining 1:1 conversion from BID TAC to OD TAC monitored C_trough_ instead of doing a full AUC_0-24_ and showed that the C_trough_ level in OD TAC was lower than BID TAC^8–13^. Such finding suggests that the C_trough_ level in the maintenance phase of KT recipients treated with OD TAC should be set at the same level as recommendation for BID TAC, which should be within the range of 5 to 7 ng/mL^1,14–16^. Therefore, the total daily dose is generally increased by 10–15% to achieve the same C_trough_ as that of BID TAC when BID TAC is switched to OD TAC^8–10,17–21^. With this strategy of the current practice, dose adjustment based on the C_trough_ level may lead to unnecessary incrementation of OD TAC dose, and unexpectedly high TAC exposure as a consequence^4^.

As a matter of fact, several previous prospective PK studies have compared the AUC_0-24_ as well as C_trough_ of BID TAC and after 1:1 conversion to OD TAC in adult KT recipients^17,22–25^. However, there were some considerations regarding these previous reports. Most of these works were supported by pharmaceutical companies. Moreover, OD TAC dose adjustment after conversion was allowed in many studies, which resulted in an increase in mean TAC dosage at the end of the study. The values of equivalence ratio used in some studies were between 0.8 and 1.25 while the most appropriate values utilized in monitoring the drug with a narrow therapeutic index such as TAC should be 0.9 and 1.11. In addition, the findings from these studies are controversial. Therefore, there is a need to assess if 1:1 conversion from BID TAC to OD TAC without subsequent dose adjustment could effectively yield comparable AUC_0-24_ levels or not.

For this intensive PK study, we applied the paradigm of bioequivalence testing to the narrow therapeutic index drugs using all of the PK parameters to compared the AUC_0-24_ and other PK parameters, before and after switching from BID TAC to OD TAC using a 1:1 dose conversion without subsequent dose adjustment in stable KT recipients. The newly calculated value of C_trough_ for OD TAC was identified. By using TAC concentrations at multiple time points instead of a single time point concentration to improve the predictive power of the C_trough_ to estimate the AUC_0-24_, we aimed to propose abbreviated AUC_0-24_ equations that would accurately predict AUC_0-24_ in our study population. The roles of CYP3A5 genotypic polymorphism on PK parameters following the 1:1 conversion without subsequent dose adjustment were also evaluated.

## Methods

### Study design and patients

A single center, open-labeled PK study was conducted at the King Chulalongkorn Memorial Hospital. The patients were consecutively enrolled from our kidney transplant clinic. The inclusion criteria were: (1) KT recipients aged ≥ 18 years, (2) on BID TAC (Prograf, Astellas, Tokyo, Japan) with mycophenolate mofetil (MMF; Cellcept, Roche, Basel, Switzerland) or enteric coated-mycophenolate sodium (EC-MPS; Myfortic, Novartis, Basel, Switzerland) and prednisolone, (3) had low to moderate risk for acute rejection^1^, (4) have stable kidney function (baseline serum creatinine < 3.0 mg/dL), and (5) had KT ≥ 6 months. Patients with a history of rejection or active infections were excluded from the study.

### Sample size

The sample size was estimated based on one of the bioequivalence criteria for drugs with a narrow therapeutic index, with a 90% confidence interval (90% CI) for the geometric mean ratio (GMR) of OD TAC/BID TAC falling within the range of 0.9–1.11^20^. Assuming log-normally distributed data with GMR of 1 in paired measurements, a correlation between the BID and OD AUC_0-24_ of 0.45 and a pooled coefficient of variation of 15%^20^, 19 participants would provide 80% power for the equivalence test using the two one-sided test approach, with a significance level of 0.05^26,27^. We increased the sample size by 5% to account for potential loss of the participants during the follow-up period. Sample size calculations were performed using SAS 9.4 (Cary, NC, USA).

### Tacrolimus measurement

KT recipients on stable doses of BID TAC and had C_trough_ between 5 to 7 ng/mL^14,15,28,29^ were admitted to the Chulalongkorn Clinical Research Center (CRC) for a 24-h PK study. Serial whole blood samples were collected immediately before administration (pre-dose), and at 0.5, 1, 2, 3, 4, 6, 8, 12, 12.5, 13,14, 15, 16, 18, 20, and 24 h after dose administration for patients taking BID TAC^30^. The patients were then switched from BID TAC to OD TAC at a ratio of 1:1 mg for 4 weeks to achieve a steady state without any subsequent dosage adjustment. Blood samples for OD TAC were obtained at pre-dose, 1, 2, 3, 4, 6, 9, 12, 15, and 24 h after dose administration^10^. The BID TAC was administered at 7:00 and 19.00 while The OD TAC dose was at 7.00. Other medications apart from TAC, including known CYP450 interaction medications were maintained at the same dose throughout the study. All patients were given a standard calorie-controlled meal that was served at the same time during the intensive PK days to minimized the effects of food on the TAC absorption^31^ All three meals were scheduled at 8.00, 12.00, and 20.00.

TAC whole blood concentrations were measured by a chemiluminescent microparticle immunoassay (ARCHITECT^®^ tacrolimus assay, ABBOTT Park, IL, USA) using 2 mL of whole blood from EDTA tubes. Each blood sample was stored at 4 to 6 °C until the assay was performed on the following day. A linear trapezoidal method was used to calculate the AUC_0-24_.

### Outcomes

The primary outcome was the AUC_0-24_ of both formulations after the 1:1 conversion. Secondary outcomes were other PK parameters, abbreviated AUC equations of OD TAC, the incidence of adverse reactions, and allograft function by estimated glomerular filtration rate (eGFR by CKD-EPI) at 1 and 3 months after conversion.

### Statistical analysis

The following PK parameters were determined utilizing non-compartmental methods: AUC_0-24_, C_trough_, C_max_, and time to maximum concentration. The data were analyzed by SPSS statistics version 18.0 (SPSS Inc., Chicago, Illinois, USA) and Stata 16.1 (StataCorp, College Station, TX). Descriptive statistics were used to summarize the participant characteristics at the first intensive PK assessment (baseline). The data that have been Ln-transformed such as AUC_0-24_, C_max_, and C_trough_ are reported as geometric mean (% coefficient of variation [%CV]), and time to C_max_ as median (IQR). Generalized estimating equations were utilized to calculate the GMR value of AUC_0-24_, C_trough_, and C_max_ in the OD TAC arm against the BID TAC arm as a reference with 90% CI. P-values were calculated based on 95% CI. Comparisons between the time to C_max_ were performed using a Wilcoxon sign rank test. For both BID TAC and OD TAC forms, linear regression models were utilized to assess the proportion of the variance in AUC_0-24_ explained by plasma concentrations at single time point, or combinations of time points using the R^2^ or adjusted R^2^ as appropriate.

### Ethics approval

The study was registered in the Thai Clinical Trials Registry (TCTR20210715002). All procedures in this study were approved by the Institutional Review Board of the Research Ethics Review Committee for Research Involving Human Research Participants, Health Sciences Group, Faculty of Medicine, Chulalongkorn University (Institutional Review Board number 538/62), in compliance with the ethical principles described in the Declaration of Helsinki and its later amendments. All participants provided written informed consent before enrollment into the study.

## Results

Twenty patients [mean (± SD) age was 46 (± 12.1) years; 60% were males] completed the study. The mean body mass index (BMI) was 22.8 (± 3.95) kg/m^2^. Median time since transplantation was 18.5 (IQR = 11.6–36.6) months. Baseline serum creatinine was 1.34 (± 0.32) mg/dL. Median total daily TAC dose was 0.058 (IQR = 0.038–0.096) mg/kg/day (Table 1). Fourteen participants (70%) were on statin, and 9 (45%) were on diltiazem, a calcium channel blocker. All patients received constant doses of sulfamethoxazole trimethoprim and acyclovir.

**Table 1:** Characteristics of study participants at first intensive PK assessment.

Variables	Value
Age in years, mean (± SD)	46 (± 12.1)
Gender, male/female, n (%)	12 (60%)/8 (40%)
Body weight, kg, mean (± SD)	61.6 (± 2.86)
BMI, kg/m^2^, mean (± SD)	22.8 (± 3.95)
Type of kidney transplant, DKT/LKT, n (%)	13 (65%)/7 (35%)
**HLA mismatch, n (%)**	
0	4 (20%)
1–5	15 (75%)
6	1 (5%)
**PRA, n (%)**	
0–10	19 (95%)
11–50	1 (5%)
≥ 50	0 (0%)
Duration after transplantation, months, median (IQR)	18.5 (11.6–36.6)
**Etiology of ESRD, n**	
DN	4
CGN, IgAN	3
Obstructive uropathy	1
Analgesic nephropathy	1
Hypertension	2
ADPKD	1
Unknown	8
Creatinine, mg/dL, mean (± SD)	1.34 (± 0.32)
eGFR CKD-EPI, mL/min/1.73 m^2^, mean (**± **SD)	60.7 (± 16.43)
Hemoglobin, g/dL, mean (± SD)	13.4 (± 1.27)
Albumin, g/dL, mean (± SD)	4.5 (± 0.22)
**CYP3A5 polymorphism, n (%)**	
Expressors [*1/*1, *1/*3]	12 (60%)
Non-expressors [*3/*3]	8 (40%)
**Total daily dose of tacrolimus**	
mg/day, median (IQR)	4.0 (2.38–5.75)
mg/kg/day, median (IQR)	0.058 (0.038–0.096)
Dose in expressors [*1/*1, *1/*3], mg/day, median (IQR)	5.5 (4.5–7)
Dose in non-expressors [*3/*3], mg/day, median (IQR)	1.75 (1.25–2.75)
Mean BID TAC C_trough_, ng/mL, mean (**± **SD)	6.03 (± 1.49)

SD: standard deviation; HLA: human leukocyte antigens; DKT: deceased donor kidney transplantation; LKT: living donor kidney transplantation; PRA: panel reactivity antibody; DN: diabetic nephropathy; CGN: chronic glomerulonephritis; IgAN: immunoglobulin A nephropathy; ADPKD: autosomal dominant polycystic kidney disease; eGFR CKD-EPI: estimated glomerular filtration rate by chronic kidney disease epidemiology collaboration equation; CYP3A5: cytochrome P450 family 3 subfamily A member 5; BID: twice daily; TAC: tacrolimus.

**Figure 1:**
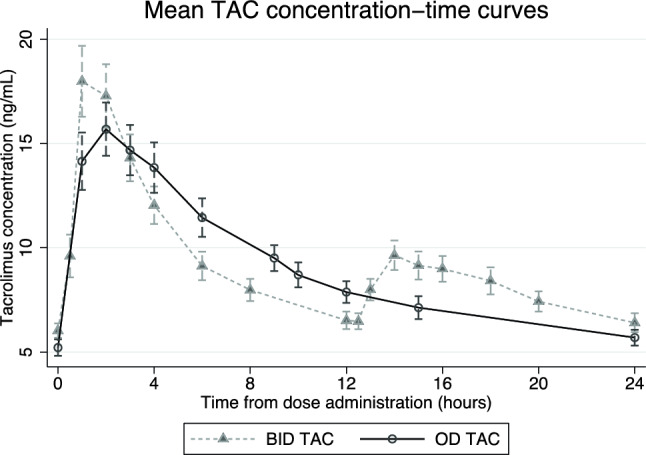
The mean tacrolimus concentration–time curves of both OD TAC and BID TAC.

The concentration–time curves of OD TAC and BID TAC are shown in Fig. 1. The geometric mean (%CV) AUC_0-24_ of OD TAC and BID TAC were 205.16 (36.4%) and 210.3 (32.5%) ng/mL × h, respectively (Table 2). The GMR (90%CI) of the AUC_0-24_ for OD TAC versus BID TAC was 0.98 (90%CI 0.91–1.04), which fell within the range of equivalence ratio. The geometric mean (%CV) C_trough_ of OD TAC and BID TAC were 5.43 (33.1%) and 6.09 (34.6%) ng/mL, respectively. The GMR of C_trough_ of OD TAC versus BID TAC was 0.89 (90% CI 0.82–0.98), which fell outside the equivalence ratio, indicating that, at the same AUC_0-24_ exposure, the C_trough_ of OD TAC was lower than the C_trough_ of BID TAC. The geometric mean (%CV) C_max_ of OD TAC and BID TAC were 15.43 (42.0%) and 18.53 (44.3%) ng/mL, respectively, with a GMR of 0.83 (90% CI 0.78–0.89) which also fell outside the equivalence ratio.

**Table 2:** Pharmacokinetic parameters of BID TAC and OD TAC and GMR for the OD versus the BID regimen.

Parametersgeometric mean (%CV)	BID TAC	OD TAC	GMR (90%CI)	p-value
AUC_0-24_, ng/mL × h	210.3 (32.5)	205.16 (36.4)	0.98 (0.91–1.04)	0.53
C_trough_, ng/mL	6.09 (34.6)	5.43 (33.1)	0.89 (0.82–0.98)	0.04
C_max_, ng/mL	18.53 (44.3)	15.43 (42.0)	0.83 (0.78–0.89)	< 0.001
Median (IQR) time to C_max_, hours	2 (1–2)	2 (1–3.5)	–	0.25

AUC_0-24_: 24-h area under the concentration–time curve; C_trough_: trough level; minimum whole-blood concentration; C_max_: maximum whole-blood concentration.

**Figure 2:**
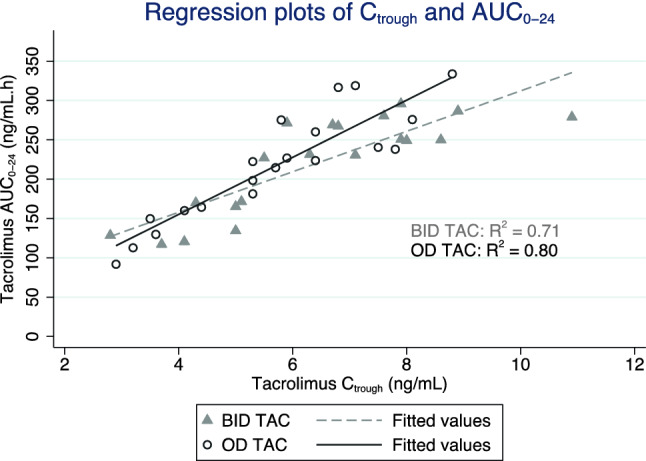
Regression plot of C_trough_ and AUC_0-24_. The equation for BID TAC is AUC_0-24_ = 55 + 25.7(C_trough_) (R^2^ = 0.71). The equation for OD TAC is AUC_0-24_ = 10 + 36.2(C_trough_), (R^2^ = 0.80).

There was a good correlation between C_trough_ and AUC_0-24_ in both BID TAC (R^2^ = 0.71) and OD TAC (R^2^ = 0.80) (Fig. 2). However, the equations for AUC prediction by C_trough_ derived from the regression plot of BID TAC and OD TAC were different. The equation for AUC_0-24_ prediction by using the C_trough_ of BID TAC was AUC_0-24_ = 55 + 25.7(C_trough_), while the equation for OD TAC was AUC_0-24_ = 10 + 36.2(C_trough_).

Since OD TAC and BID TAC have different formulations and PK, thus, OD TAC should have its own specific target C_trough_ level and it should not be the same as the target C_trough_ level of BID TAC. According to the targeted C_trough_ level of BID TAC (5 to 7 ng/mL), the AUC_0-24_ can be calculated from the equations presented in Fig. 2 which was between 183.5 and 234.9 ng/mL × h. By aiming for the same level of AUC_0-24_ as BID TAC, the new target C_trough_ of OD TAC can be calculated from the OD TAC equation and ranged from 4.8 to 6.2 ng/mL.

**Figure 3:**
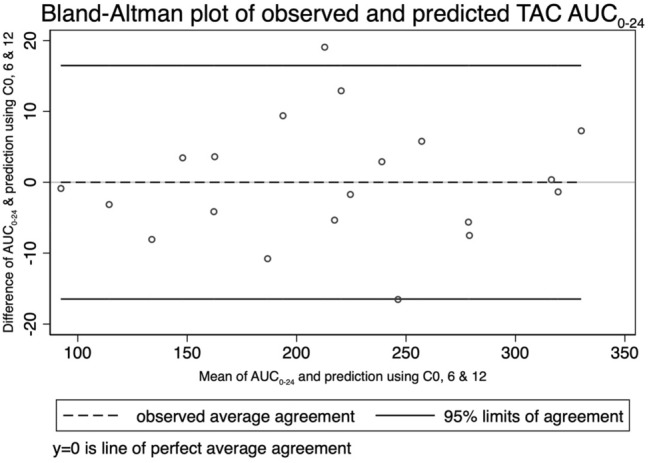
Bland–Altman plot between observed and predicted TAC AUC_0-24_ by C0, C6, and C12 equation.

To achieve more accuracy than the single timepoint monitoring but less complicated measurement than the full AUC_0-24_, the abbreviated AUC_0-24_ of OD TAC and AUC_0-12_ of BID TAC derived from two- and three-time point regression equations were detailed in Tables 3 and 4, respectively. The abbreviated AUC_0-24_ equation derived from C0, C6, and C12 had the highest correlation with AUC_0-24_. A Bland–Altman plot of AUC_0-24_ is depicted in Fig. 3. The average difference between the linear prediction based on C0, C6, and C12 and the actual AUC_0-24_ was 0.0 (SD ± 8.4) ng/mL × h, with a 95% limit of agreement extending from − 16.47 to 16.47 ng/mL × h. The scatter of the individual points showed no evidence of bias across the range of the AUC_0-24_. Lin’s concordance correlation coefficient was 0.99.

**Table 3:** The proportion of variance in OD TAC AUC_0-24_ is explained by single TAC levels, or combinations of TAC levels at multiple time points.

Time point	Equations	R^2^
C0 (0 h)		0.80
C1 (1 h)		0.58
C2 (2 h)		0.68
C3 (3 h)		0.87
C4 (4 h)		0.91
C6 (6 h)		0.91
C9 (9 h)		0.92
C10 (10 h)		0.92
C12 (12 h)		0.96
C15 (15 h)		0.92
C24 (24 h)		0.80
C0, C2	AUC = 24.68(C0) + 4.40(C2) + 18.87	0.85
C0, C1, C2	AUC = 26.60(C0) + 3.82(C2) + 0.25(C2) + 19.7	0.86
C0, C4	AUC = 11.64(C0) + 8.90(C4) + 32.68	0.92
C0, C3, C4	AUC = 10.47(C0) + 2.09(C3) + 7.23(C4) + 31.05	0.92
C0, C4, C6	AUC = 10.13(C0) + 3.42(C4) + 8.02(C6) + 24.59	0.94
C0, C4, C10	AUC = 4.67(C0) + 5.29(C4) + 12.62(C10) + 9.15	0.97
C0, C6, C9	AUC = 6.20(C0) + 7.02(C6) + 11.00(C9) − 0.66	0.97
C0, C6, C12	AUC = 0.97(C0) + 5.79(C6) + 18.97(C12) − 4.26	0.98

**Table 4:** The proportion of variance in BID TAC AUC_0-12_ is explained by single TAC levels, or combinations of TAC levels at multiple time points.

Time point	Equations	R^2^
C0 (0 h)		0.74
C0.5 (0.5 h)		0.35
C1 (1 h)		0.53
C2 (2 h)		0.82
C3 (3 h)		0.92
C4 (4 h)		0.88
C6 (6 h)		0.79
C8 (8 h)		0.85
C12 (12 h)		0.77
C1, C3, C6	AUC_0-12_ = 1.23(C1) + 3.88(C3) + 4.40 (C6) + 6.76	0.99
C0, C2	AUC_0-12_ = 12.25(C0) + 2.94(C2) − 0.025	0.90
C0, C2, C3	AUC_0-12_ = 5.22(C0) + 0.98(C2) + 4.69(C3) + 9.32	0.94
C0, C2, C4	AUC_0-12_ = 1.90(C0) + 2.05(C2) + 5.88(C4) + 10.54	0.95
C0, C3, C4	AUC_0-12_ = 1.99(C0) + 4.64(C3) + 2.74(C4) + 13.15	0.94
C0, C3, C6	AUC_0-12_ = 2.35(C0) + 4.95(C3) + 3.23(C6) + 10.18	0.95
C0, C4, C6	AUC_0-12_ = 3.17(C0) + 6.14(C4) + 2.48(C6) + 8.86	0.91
C2, C3	AUC_0-12_ = 0.65(C2) + 6.46(C3) + 20.93	0.93
C2, C4	AUC_0-12_ = 2.07(C2) + 6.21(C4) + 14.65	0.96
C2, C3, C4	AUC_0-12_ = 1.83(C2) + 0.83(C3) + 5.55(C4) + 14.41	0.96
C2, C3, C6	AUC_0-12_ = 1.47(C2) + 3.15(C3) + 4.46(C6) + 13.46	0.96

In addition, we further investigated the effects of CYP3A5 genotypic polymorphism on AUC_0-24_ and C_trough_ after converting from BID TAC to OD TAC. The *CYP3A5* gene alleles were identified in the whole blood by real-time reverse transcription polymerase chain reaction by using forward and reverse primers (F5′-CAT GAC TTA GTA GAC AGA TGA-3′, R 5′-GGT CCA AAC AGG GAA GAA ATA-3′). A fluorescent TaqMan probe was utilized to identify the allelic variant of *CYP3A5* (rs776746). The patients were then categorized according to CYP3A5 genotypic polymorphism: (1) expressor (CYP3A5 *1/- or CYP3A5 *1/*3) and (2) non-expressor (CYP3A5 *3/*3).

**Figure 4:**
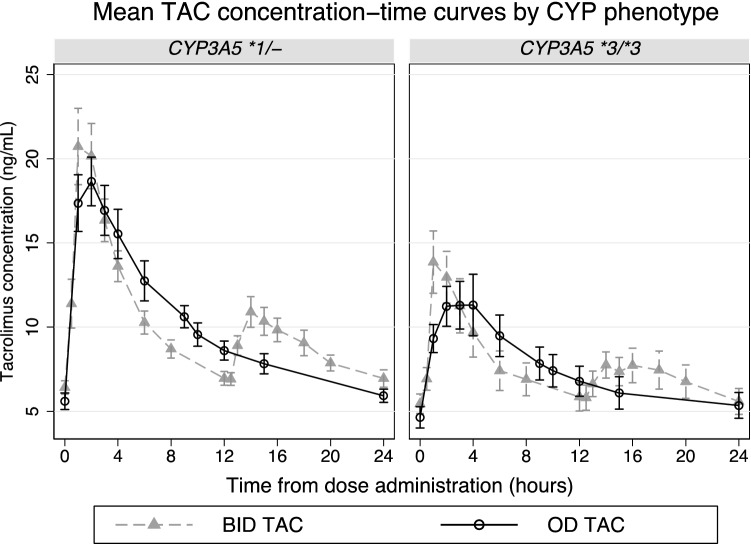
The mean (± SE) tacrolimus concentration–time curves by *CYP3A5* genotype of both BID TAC and OD TAC.

Twelve of the 20 patients were CYP3A5 expressor while the remaining patients were non-expressor. In the CYP3A5 expressor group, the geometric means (%CV) of AUC_0-24_ were 234.5 (26.3%) and 238.5 (23.5%) ng/mL × h for OD TAC and BID TAC, respectively (Table 5). The GMR (90%CI) was 0.98 (0.91–1.05). The geometric mean (%CV) of C_trough_ for OD TAC and BID TAC were 5.77 (24.7%) and 6.74 (25.8%) ng/mL, respectively, with a GMR (90%CI) of 0.86 (0.79–0.93) which fell outside the equivalence ratio (Fig. 4). In the CYP3A5 non-expressor group, the geometric mean (%CV) of AUC_0-24_ for OD TAC and BID TAC were 167.9 (41.1%) and 174.1 (35.8%) ng/mL × h, respectively, with a GMR (90%CI) of 0.96 (0.85–1.09). The geometric means (%CV) of C_trough_ of OD TAC and BID TAC were 4.96 (43.6%) and 5.21 (41.8%) ng/mL, respectively, with a GMR C_trough_ of 0.95 (0.80–1.13). The GMR OD TAC/BID TAC of both AUC_0-24_ and C_trough_ in the non-expressor group fell within the equivalence ratio.

**Table 5:** AUC_0-24_ and C_trough_ with GMR (90%CI) for the OD versus the BD regimen, by CYP3A5 expression.

Variablesgeometric mean (%CV)	BID TAC	OD TAC	GMR (90%CI)	p-value
CYP3A5 expressor (CYP3A5 *1/-, n = 12)				
AUC_0-24_, ng/mL × h	238.5 (23.5)	234.5 (26.3)	0.98 (0.91–1.05)	0.70
C_trough_, ng/mL	6.74 (25.8)	5.77 (24.7)	0.86 (0.79–0.93)	0.003
AUC_0-24_/dose, ng/mL × h**/**mg/kg/day	1,749 (74.6)	1,719.9 (79.0)	0.98 (0.90–1.07)	0.70
CYP3A5 non-expressor (CYP3A5 *3/*3, n = 8)				
AUC_0-24_, ng/mL × h	174.1 (35.8)	167.9 (41.1)	0.96 (0.85–1.09)	0.62
C_trough_, ng/mL	5.21 (41.8)	4.96 (43.6)	0.95 (0.80–1.13)	0.64
AUC_0-24_/dose, ng/mL × h**/**mg/kg/day	5,978.0 (24.7)	5,763.2 (15.6)	0.96 (0.83–1.11)	0.62

**Figure 5:**
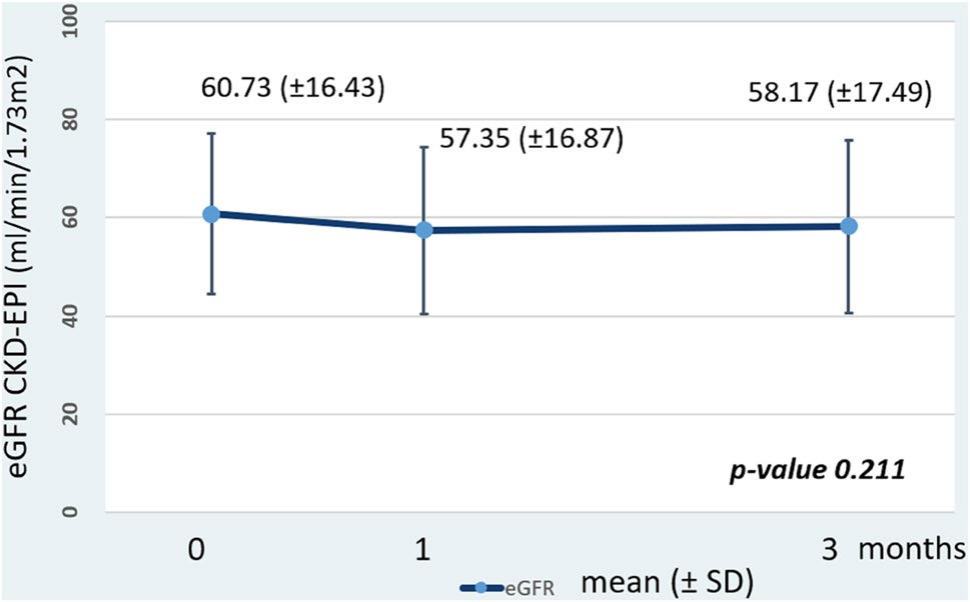
Allograft function by eGFR CKD-EPI at before conversion, one month, and 3 months after conversion; p-value by repeated ANOVA. (eGFR CKD-EPI; estimated glomerular filtration rate by chronic kidney disease epidemiology collaboration equation).

There were no major adverse reactions including acute rejection, during the dose conversion period and 3 months after the conversion period. The CKD-EPI eGFR remained stable throughout the study period (Fig. 5).

## Discussion

The results in the present PK study have demonstrated that 1:1 dose conversion in drug with a narrow therapeutic index such as TAC, from BID TAC to OD TAC without subsequent dose adjustment in stable adult KT recipients who received constant immunosuppressive regimens had a GMR of AUC_0-24_ of OD TAC/BID TAC of 0.98 (90%CI 0.91–1.04) which fell within the range of equivalence ratio (90%CI = 0.9–1.1) while GMR of C_trough_ was 0.89 (90%CI = 0.82–0.98) which fell outside the equivalence ratio. The regression plot of AUC_0-24_ and C_trough_ found that, at the same AUC_0-24_ level, OD TAC had lower C_trough_ level compared with BID TAC. Patients in the CYP3A5 expressor group exhibited comparable AUC_0-24_ despite significantly decreased C_trough_ after 1:1 conversion while the non-expressor group showed similar AUC_0-24_ and C_trough_.

A comprehensive PK data from all previous prospective PK studies using 1:1 conversion from BID TAC to OD TAC are illustrated in Table 6. In an earlier study by Alloway et al., 20 of 66 patients who had completed PK profiles had TAC dose adjustment during the PK studies for various reasons^17^. Of note, the values of equivalence ratio used in the study were 0.8–1.25. Despite TAC dose adjustment, the GMR value of AUC_0-24_ between OD TAC and BID TAC was 0.95 which fell within the equivalence ratio while that of C_trough_ was 0.87 which fell outside the equivalence ratio of narrow therapeutic index (Table 6). In another PK study conducted by Midtvedt et al., the GMR values of AUC_0-24_ and C_trough_ of OD TAC and BID TAC were 0.82 and 0.81, respectively^23^. Moreover, the study allowed subsequent dose adjustments during the following 2–3 weeks post conversion in order to keep the C_trough_ concentration within 5–10 ng/mL. Likewise, a study conducted by Stifft et al., the TAC doses were subsequently increased by 1, 1.5, and 2 mg to reach a C_trough_ greater than 4.0 ng/mL^25^. The GMR value of AUC_0-24_ was 0.98 while that of C_trough_ was 0.89 which fell outside the equivalence ratio. Since all of these three PK reports were 1-way conversion studies, van Hooff et al., utilized a 4-period crossover replicate study design in 60 KT patients^24^. Although TAC dose adjustments were prescribed in some of the patients, the analyses were performed in patients without dose modifications. The precise results showed that the values of GMR of AUC_0-24_ and C_trough_ between both TAC formulations fell within the equivalence ratio. However, it should be noted that the results from these PK studies of 1:1 dose conversion from BID TAC to OD TAC should be interpreted cautiously because there were subsequent dose adjustments and most of these earlier reports were pharmaceutical company-sponsored studies. In addition, the results were inconsistent across the studies.

**Table 6:** Prospective study of 1:1 mg conversion from BID to OD TAC in stable adult kidney transplant recipients with AUC_0-24_ monitoring.

Study	Alloway^17^	Midtvedt^23^	van Hooff^24^	Stifft^25^	The present study
Population	N = 66 Post-transplant > 6mo	N = 20 Post-transplant > 6mo	N = 60 Post-transplant > 6mo	N = 40 Post-transplant > 15mo	N = 20 Post-transplant > 6mo
Ethnicity	Mainly Caucasian	Caucasian	Mainly Caucasian	Caucasian	Asian
Trial design	Open label, 1:1 mg conversion	Open label, 1:1 mg conversion	Open label, 1:1 mg conversion	Open label, 1:1 mg conversion	Open label, 1:1 mg conversion
Dose adjustments allow	Yes	Yes (no dose adjustment in 18 patients)	Yes (analysis was made in patients without dose adjustment)	Yes	No
Pharmaceutical company sponsored	Yes	No	Yes	Yes	No
C_trough_ BID vs OD TAC	Mean C_trough_ BID TAC = 6.6 ng/mL Mean C_trough_ OD TAC = 5.7 ng/mL Equivalence ratio = 0.87 (90%CI 0.83–0.92)	Mean C_trough_ BID = 6.6 ± 2.9 ng/mL Mean C_trough_ OD TAC = 5.4 ± 1.4 ng/mL	Mean C_trough_ BID TAC = 6.60 ng/mL Mean C_trough_ OD TAC = 7.26 ng/mL	Mean C_trough_ BID TAC = 7.4 (7.0–7.7) ng/mL Mean C_trough_ OD TAC = 6.6 (6.2–7.0) ng/mL (p = 0.003)	Mean C_trough_ BID = 6.09 ng/mL (CV 34.6%) Mean C_trough_ OD TAC = 5.43 ng/mL (CV 33.1%) GMR = 0.89 (0.82–0.98)
AUC BID vs OD TAC	AUC_0-24_ BID TAC = 202.5 ng/mL × h AUC_0-24_ OD TAC = 192.3 ng/mL × h Ratio = 0.94 (90%CI 0.90–0.99)	AUC_0-24_ BID TAC = 265 ± 112 ng/mL × h AUC_0-24_ OD TAC = 218 ± 47 ng/mL × h	AUC_0-24_ BID TAC = 217.75 ng/mL × h AUC_0-24_ OD TAC = 234.42 ng/mL × h	AUC_0-24_ BID TAC = 219.2 (208.1–230.9) ng/mL × h AUC 0–24 OD TAC = 213.3 (202.6–224.5) ng/mL × h (p = 0.37)	Mean AUC_0-24_ BID TAC = 210.3 ng/mL (CV 32.5%) Mean AUC_0-24_ OD TAC = 205.16 ng/mL (CV 36.4%) GMR = 0.98 (0.91–1.04)
Conclusion	After dose adjustment, PK of OD TAC was equivalent to BID TAC	C_trough_ decreased after conversion	Both AUC_0-24_ and C_trough_ of BID TAC and OD TAC are similar after conversion	After dose adjustment, AUC_0-24_ are similar, but C_trough_ was lower in OD TAC	After conversion, AUC_0-24_ are the same, but the C_trough_ of OD TAC is lower

Our findings established that in the 1:1 conversion from BID TAC to OD TAC without subsequent TAC dose adjustment, the TAC exposure remains similar despite being approximately 11% lower in the C_trough_ level (Table 2). Since the AUC_0-24_ revealed a similar level of TAC exposure after conversion, the 10% to 15% incrementation of OD TAC dose to maintain the same level of C_trough_ currently performed in routine clinical practice is not necessary. As shown in Tables 3 and 4, the equations derived from the regression plot of C_trough_ for AUC prediction (abbreviated AUC) for both BID TAC and OD TAC are different, indicating that the target C_trough_ level of OD TAC used in real clinical practice should not be the same as that of BID TAC. For a targeted level of AUC_0-24_ within the range of 180–240 ng/mL × h, the practical used C_trough_ level of BID TAC is 5 to 7 ng/mL, the C_trough_ levels specific for OD TAC should be 4.8 to 6.2 ng/mL. This should be beneficial to physicians in prescribing the dose of OD TAC and monitoring TAC exposure, particularly in the places where PK studies of TAC are not easily to performed.

As stated earlier, the abbreviated AUC of BID TAC is more accurate than C_trough_ and is less complicated than AUC_0-12_ or full AUC_0-24_ in therapeutic drug monitoring for KT recipients^28,32^. Since BID TAC and OD TAC have different concentration profiles, the abbreviated AUC_0-24_ equations of BID TAC should not be used to predict AUC_0-24_ for OD TAC. In this regard, there is only one study of abbreviated AUC_0-24_ for OD TAC published which was conducted in pediatric KT recipients^33^. Herein, our full AUC_0-24_ study of OD TAC provides abbreviated AUC equations derived from stable adult KT recipients (Table 3). Physicians can choose one of these equations to suit clinical practice by considering the number and timing of the blood draws. Moreover, for physicians who still mainly use AUC_0-12_ for BID TAC adjustment, our study also provides abbreviated AUC_0-12_ for BID TAC (Table 4).

Of note, individual TAC PK can be affected by several factors, including hemoglobin levels, serum albumin, drug interactions, ABCB1 or MDR1 gene expression, and CYP3A5 genotypic polymorphism. The CYP3A5 non-expressor recipient (*3/*3 genotype) requires a lower dose, while the expressor recipient (*1/*1 and *1/*3) needs a greater dose to attain the target TAC levels^34–36^. Of interest, more than 80% of the Caucasians are non-expressor while approximately 50% of Asians are non-expressor^37^. This disparity may affect the PK profile of TAC among different ethnicities.

There are sparse data regarding the role of CYP3A5 genotypic polymorphism on PK profile in 1:1 conversion from BID TAC to OD TAC. Following conversion from BID TAC to OD TAC, earlier retrospective PK studies by Wehland et al., and Jonge et al. demonstrated that C_trough_ was only significantly reduced in non-expressors^9,38^. Unfortunately, both studies had a limited number of patients who were CYP3A5 expressor. In the study by Wehland et al., non-expressors were younger and more likely to receive kidneys from living donors, and also tended to have better renal function. Furthermore, AUC_0-24_ was not performed in both PK studies. A following prospective PK study by Glowacki et al., showed that the C_trough_ levels were comparable in the non-expressor group but were significantly lower in OD TAC when compared with BID TAC in the expressor group^22^. The AUC_0-24_ values were comparable after 1:1 conversion from BID TAC to OD TAC in both expressor and non-expressor groups. However, when the values of AUC_0-24_ were adjusted by TAC dose, the dose-adjusted AUC_0-24_ in OD TAC were slightly but significantly lower than BID TAC in both CYP3A5 groups. Our study showed that AUC_0-24_ and dose-adjusted AUC_0-24_ were similar for both expressor and non-expressor groups following 1:1 conversion from BID TAC to OD TAC without subsequent dose adjustment (Table 5). The discrepancies between the present PK study and that by Glowacki et al., are still inconclusive. The aim of study from Glowacki et al., was to compare the PK profiles between expressor and non-expressor groups. The included participants were categorized into two groups at the start of the study while our work examined the roles of CYP3A5 in the second part of the study, possibly resulting in a less biased study. Nonetheless, both studies had a small sample size. Future studies with a larger sample size are warranted.

The strength of this study was that the 1:1 conversion from BID TAC to OD TAC was strictly controlled. All PK studies were conducted at the Clinical Research Center which provided perfect facilities for the clinical study. However, there were some limitations in this study. First, this was a small, cross-sectional study conducted specifically in Asian KT recipients. Second, 99% of the variability in AUC_0-24_ was accounted for in the linear equation using C0, C6 and C12 as predictor covariates, and the 95% limits of agreement between the actual AUC and predicted AUC were within a range of ± 16.5 ng/mL × h. However, it is unknown whether this equation will perform equally well in an external validation cohort or not. Additional prospective studies assessing the safety and efficacy of the specific target level for OD TAC are crucially needed.

## Conclusions

Conversion from BID TAC to OD TAC with a 1:1 daily dose without subsequent dose adjustment is appropriate and provides similar TAC exposure regardless of CYP3A5 genotypic polymorphism. Despite the decrease in C_trough_ of OD TAC, increasing the dose to aiming the same C_trough_ level as BID TAC is not necessary. The pharmacokinetics of both OD TAC and BID TAC are different. The differences in the target C_trough_ are acceptable when the AUC_0-24_ of both drugs are comparable.

## Abbreviations

AUC: Area under the concentration–time curve
BID TAC: Twice daily tacrolimus
C_trough_: Trough level, minimum whole-blood concentration
C_max_: Maximum whole-blood concentration
GMR: Geometric mean ratio
OD TAC: Once daily tacrolimus
TAC: Tacrolimus
Tmax: Time to achieve maximum whole-blood concentration
